# *FAM83D*, a microtubule-associated protein, promotes tumor growth and progression of human gastric cancer

**DOI:** 10.18632/oncotarget.20157

**Published:** 2017-08-10

**Authors:** Minlu Huang, Xinjie Ma, Hongpeng Shi, Lei Hu, Zhiyuan Fan, Li Pang, Fan Zhu, Xiao Yang, Wei Xu, Binya Liu, Zhenggang Zhu, Chen Li

**Affiliations:** ^1^ Shanghai Key Laboratory of Gastric Neoplasms, Department of Surgery, Shanghai Institute of Digestive Surgery, Ruijin Hospital, Shanghai Jiao Tong University School of Medicine, Shanghai 200025, People’s Republic of China

**Keywords:** FAM83D, gastric cancer, proliferation, HMMR, TPX2

## Abstract

*FAM83D*, a microtubule-associated protein (MAP), is overexpressed in diverse types of human cancer. The expression and critical role of *FAM83D* in human gastric cancer (GC), however, remains largely unknown. Here, we conducted molecular, cellular and clinical analyses to evaluate the functional link of *FAM83D* to GC. *FAM83D* expression was elevated in gastric tumors, and its expression strongly correlated with lymph node metastasis and TNM stage. In addition, over-expression of *FAM83D* in GC cell lines enhanced cell proliferation, cycle progression, migration, invasion, as well as tumor growth and metastatic dissemination *in vivo*. Furthermore, *FAM83D* exhibited a strong cell cycle correlated expression. The knockdown of *FAM83D* inhibited the regrowth of microtubules in GC cells. FAM83D was co-immunoprecipitated with HMMR, TPX2, and AURKA, a set of drivers of mitosis progression. Taken together, our results demonstrate *FAM83D* as an important player in the development of human gastric cancer, and as a potential therapeutic target for the treatment of cancer.

## INTRODUCTION

Gastric cancer (GC) is one of the leading causes of cancer-related death worldwide [1–3]. The current treatment of this disease, however, is still largely relying on the surgical removal and/or chemotherapies due to a shortage of target therapies. Thus, understanding how GC is regulated at genetic, molecular and signaling levels will provide promising targets for the development of novel therapeutic agents to improve current treatment.

Recently, Family with sequence similarity 83 member D (*FAM83D*), also known as *C20Orf129* or *CHICA*, has been implicated as a crucial player in the GC malignancy. This gene was initially identified as a novel spindle component by mass-spectrometry [4]. It contains a highly conserved domain, DUF1669, which is critical for CRAF binding and activation of MAPK signaling [5, 6]. In addition, *FAM83D* is situated at a cancer-susceptible chromosomal locus (20q), which is frequently amplified in a variety of human cancers, including gastric cancer [7–9]. Also, *FAM83D* is elevated at the protein level in human tumor tissues and is associated with poor prognosis in breast cancer and hepatocellular carcinoma [10, 6, 11, 12]. In addition, *FAM83D* is implicated in promoting tumor cell proliferation, migration, and invasion through activation of the mTOR- and MAPK- signaling pathway [6, 10]. These observations implicate *FAM83D* as a strong driver of tumor development and progression in human cancer.

**Figure 1:**
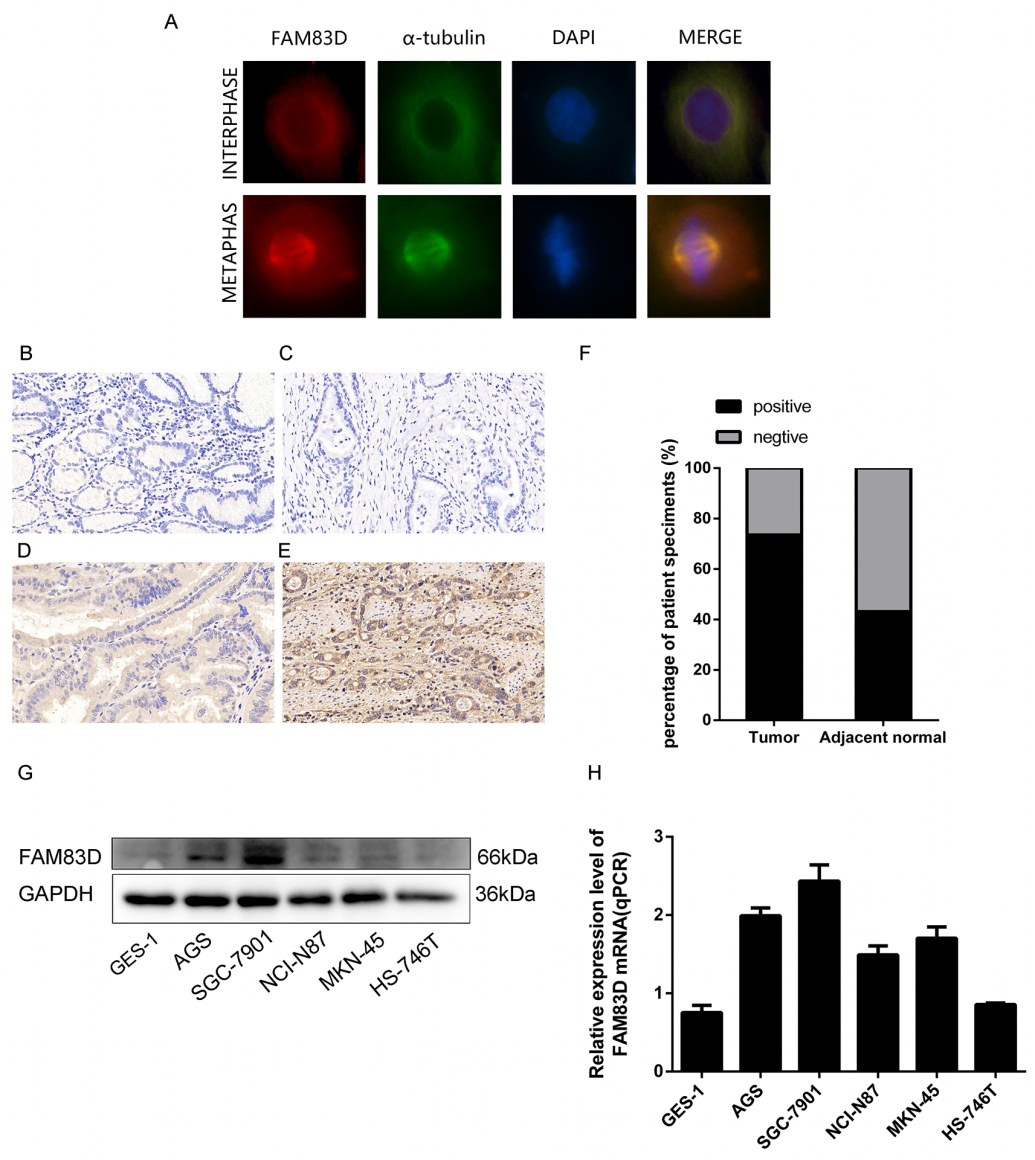
Expression of FAM83D in human gastric tumor tissues and cell lines **(A)** Immunofluorescence images of AGS cells at interphase and metaphase stained with antibodies against FAM83D (red), α-tubulin (green), and DAPI (blue). **(B)** Negative FAM83D expression in the non-tumor gastric mucosa (200x). **(C, D, and E)** Negative, weak positive and strong positive FAM83D expression in gastric cancer tissues. **(F)** Positive ratios of FAM83D expression in 102 pairs of gastric cancer tissues. **(G and H)** mRNA and protein expression of FAM83D in GC cell lines.

FAM83D protein localizes to the cytoplasm during interphase and to the spindle microtubules and poles during mitosis [4, 13] (Figure 1A). It is increased and phosphorylated upon cell upon entering the G2/M phase [13, 14], indicating its dynamic mitotic function. In line with these observations, the depletion of FAM83D impairs chromosomes aggregation on the metaphase plate and the nuclear envelope breakdown (NEBD), thereby delaying the onset of anaphase during mitosis [13, 15]. Also, *FAM83D* appears to be concomitantly decreased with a group of mitosis-related genes (PLK1, CDC25B, BUB1B, and Cyclin-F) by MiR-210 [16]. Furthermore, *FAM83D* is overexpressed in primary tumors carrying TP53 mutations, compared to those with intact TP53 [12, 17]. Together, these lines of evidence suggest that *FAM83D* impacts tumor cell proliferation or growth by controlling cell cycle progression.

At the molecular level, FAM83D appears to form a complex with DYNLL1 and HMMR, promoting proper mitotic spindle orientation, rather than directly impacting on astral microtubules [15]. In particular, HMMR, a cell surface hyaluronan receptor and mitotic spindle protein and the driver of tumor progression [18], has been implicated in the targeting of FAM83D to the mitotic spindle [15, 19]. It also appears to regulate function and mitotic location of TPX2 [19, 20], which is essential for targeting AURKA to the spindle [21, 22]. Furthermore, TPX2 (c20orf1), the targeting protein for Xklp2, regulates the organization of MTs [23]. Inhibition of HMMR or TPX2 severely impairs mitotic spindle assembly and integrity [20, 24–28]. Also, Aurora-A, known as Aurora kinase A (AURKA), promotes tumorigenesis by affecting cell cycle progression [29]. In particular, this kinase appears to stimulate timely mitotic entry through the activation of PLK1 and the Cyclin-B1/Cdk-1 complexes [30, 31]. Collectively, these observations raise a strong possibility that FAM83D coordinates cell cycle progression in human cancer cells by binding with HMMR, TPX2, and AURKA.

Based on emerging molecular and functional links of *FAM83D* to mitosis, we hypothesized that *FAM83D* promotes gastric tumor growth and progression by stimulating cell cycle progression through binding with HMMR, TPX2, and AUKRA. To test this hypothesis, we analyzed effects of *FAM83D* overexpression and knockdown on cancer cell cycle progression, tumor growth, and metastasis. Also, immunoprecipitation was carried out to evaluate the interaction between FAM83D and HMMR, TPX2 and AUKRA. In addition, IHC analyses of human tumor tissues were conducted to determine the correlation between *FAM83D* and key clinical characteristics of GC. Data from our analyses demonstrate that *FAM83D* promotes tumor growth and metastasis in gastric cancer. To a larger extent, these functions of *FAM83D* are linked to the control of cell cycle progression and correlated with MAPs. As such, our study has provided strong evidence that *FAM83D* is a key driver of tumor development and progression in gastric cancer and is a promising therapeutic target for the treatment of this disease.

## RESULTS

### Association between *FAM83D* expression and key clinicopathological parameters of human gastric cancer

To evaluate *FAM83D* expression pattern in human GC, immunohistochemistry (IHC) was performed in 102 tumor samples and matched non-tumor tissue pairs. Our data showed that 73.5% ( 75 of 102 positive) tumor samples showed positive staining for FAM83D, whereas in non-tumor tissue samples, 44 out of 102 stained positive (P<0.01) (Figure 1B-1F). Furthermore, high FAM83D upregulation was strongly correlated with an increased lymph node metastasis, advanced TNM stage (P= 0.006; P<0.001), but not with other clinical parameters (Table 1). In parallel, our analyses of the published Gene Expression Omnibus (GEO) database [32, 33] showed that *FAM83D* was also significantly higher at mRNA level in gastric cancer group than their normal counterparts (P=4.60E-05, P=0.000217). Moreover, a similar trend was detected for *FAM83D* in our analyses with human GC cell lines, including AGS, SGC-7901, NCI-87, MKN-45, HS-746T and one immortalized normal gastric epithelial cell line (GES-1) (Figure 1G-1H). Together, these data demonstrate an upregulation of *FAM83D* expression in human gastric tumors and implicate its role in the development of GC.

**Table 1:** Correlation of FAM83D expression with clinic-pathologic features

Variables	Number of cases	FAM83D immunostaining		P value
		Positive	Negative	
Gender				
male	72	50	22	0.105
female	30	25	5	
Ags(years)				
<60	44	35	9	0.235
≥60	58	40	18	
Differentiation				
well to moderate	37	28	9	0.675
poor	65	47	18	
Poortumor size				
≤5	60	46	14	0.447
>5	42	29	13	
T stage				
T1+T2	17	9	8	0.067
T3+T4	85	66	19	
Lymph node metastasis				
YES	71	58	13	0.006
NO	31	17	14	
TNM stage				
I+II	38	20	18	<0.001
III+IV	64	55	9	

### *FAM83D* drives GC cell proliferation

**Figure 2:**
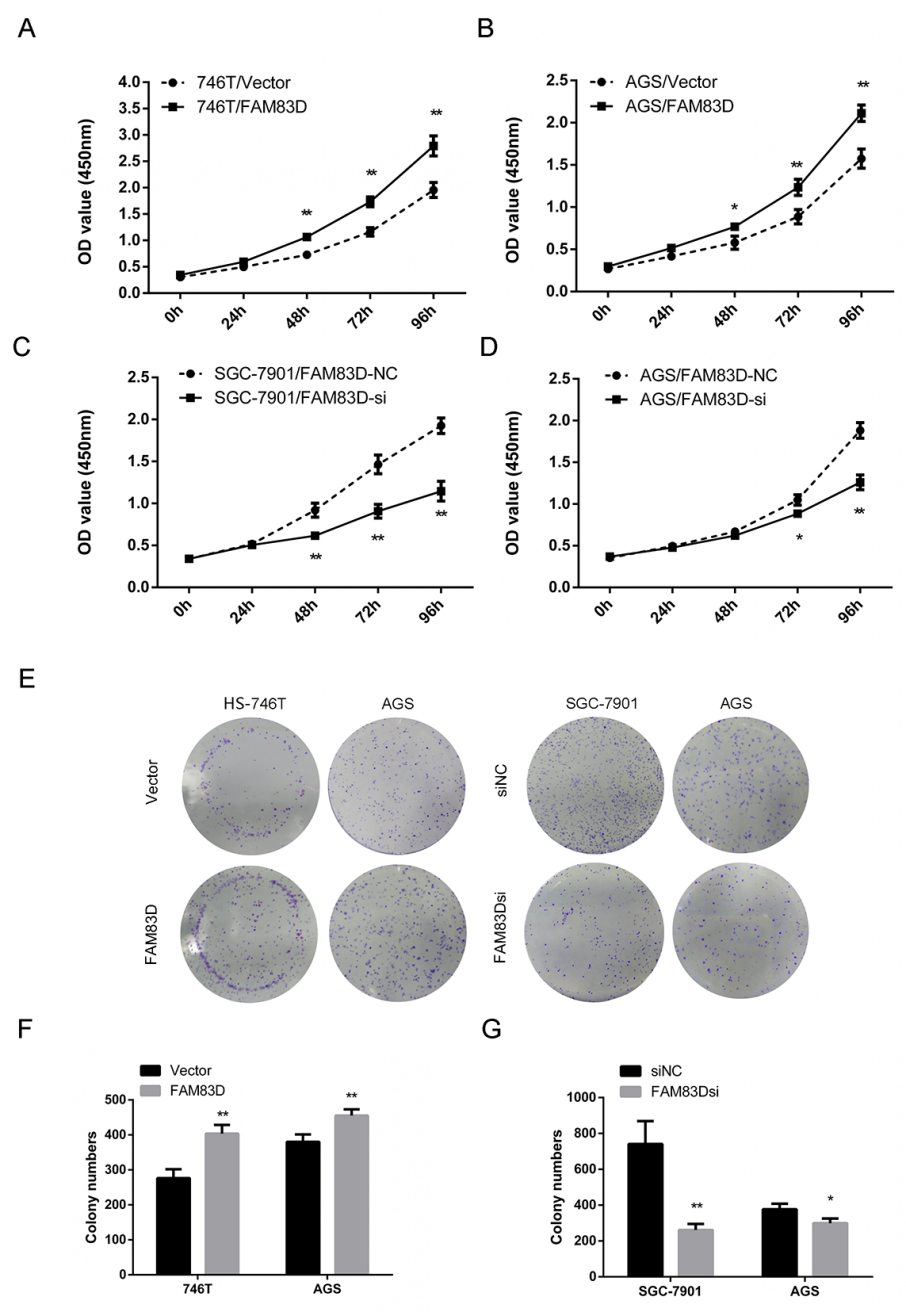
Ectopic expression of FAM83D promotes GC cell proliferation **(A** and **B)** The effect of FAM83D overexpression on HS-746T and AGS cells proliferation were measured by Cell Counting Kit-8 assay. **(C** and **D)** SGC-7901 and AGS cells transfected with FAM83D siRNA and controls at 24 h post-transfection were subjected to CCK8 assay. **(E, F,** and **G)** Photographs and histograms of colony formation assay demonstrated the number of colonies, *P < 0.05, **P < 0.01.

Based on the clinical link, we evaluated the function of *FAM83D* in human GC. As shown in Figure 2A-2B, overexpressing *FAM83D* markedly increased cell proliferation in both 746T and AGS lines, compared to each of their vector controls. Conversely, knockdown of FAM83D via three distinct RNAi oligos (Supplementary Table 1, Supplementary Figure 1B) led to a decrease in cell growth (Figure 2C-2D, Supplementary Figure 2A). In addition, a similar trend was detected for colony formation by GC cells upon overexpression or knockdown of *FAM83D* (Figure 2E-2G, Supplementary Figure 2B, P < 0.01). Together, these data demonstrated that *FAM83D* promoted cell proliferation of GC cells.

**Figure 3:**
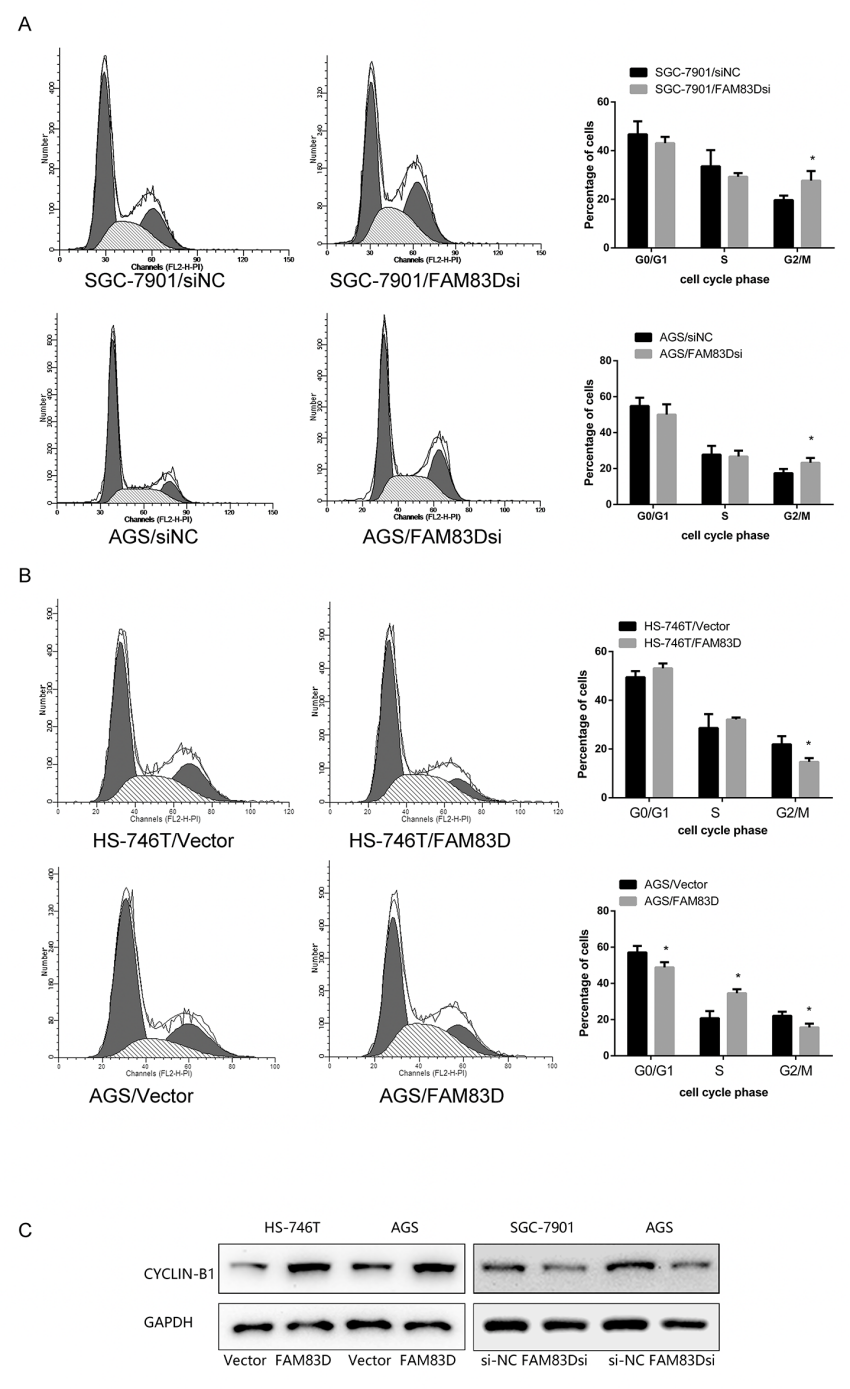
Effect of FAM83D on the cell cycle in GC cells **(A)** Flow cytometry analysis was performed on SGC-7901 and AGS cells that were transfected with FAM83D siRNA and control siRNA. **(B)** Flow cytometry analysis was performed on FAM83D-expressing HS-746T and AGS cells. **(C)** Western blot analysis of cyclin-B1.

We next tested if *FAM83D* promoted cell proliferation by impacting cell-cycle progression. As shown in Figure 3 and Supplementary Figure 2C, *FAM83D* siRNA-treated AGS and SGC-7901 cells led to a significant increase in the proportion of tumor cells at the G2-M phase (AGS: 23.26% ± 2.65% vs. 17.42% ± 2.36%, P < 0.05; SGC-7901: 27.67% ± 3.96% vs. 19.68% ± 1.86%, P < 0.05). Conversely, overexpressing *FAM83D* decreased the proportion of tumor cells in the G2/M phase. Notably, the proportions of cells at the G2/M phase for *FAM83D*-overexpression and control cells were 3.06%± 1.00% and 10.62% ± 4.32%, in AGS line, respectively (P < 0.05), and 4.17% ± 3.73% and 11.35% ± 1.81% in HS-746T cell line (P < 0.05). Interestingly, overexpressing *FAM83D* in AGS line also reduced the distribution of tumor cells in the G1/G0 phase (56.99% ± 2.27% vs. 66.50% ± 5.36%, P < 0.05). Collectively, these observations suggested that *FAM83D* promotes proliferation of GC cells by accelerating cell cycle progression through both G1/S and G2/M phases.

To further clarify the nature of the G2/M delay associated with *FAM83D* deficiency, the protein level of cyclin-B1, a hallmark of G2/M phase [34], was examined. The knockdown of *FAM83D* level led to a striking decrease of the cyclin-B1 level. In contrast, the opposite results were obtained with *FAM83D* overexpression (Figure 3C). Taken together, *FAM83D* is crucial in the promotion or development of tumor cell growth while ablation of *FAM83D* negative impacts proliferate capacity. The effect of *FAM83D* knockdown on cell apoptosis and senescence was further investigated. We detected a minimal difference in the percentage of Annexin V-positive apoptotic or β-Gal- positive senescent cells between the *FAM83D* knockdown and control groups (data not shown). Thus, *FAM83D* increases proliferation of GC cells by enhancing cell cycle progression, rather than the induction of cell apoptosis or senescence.

### *FAM83D* promotes the wound healing, migration, and invasion of GC cells

**Figure 4:**
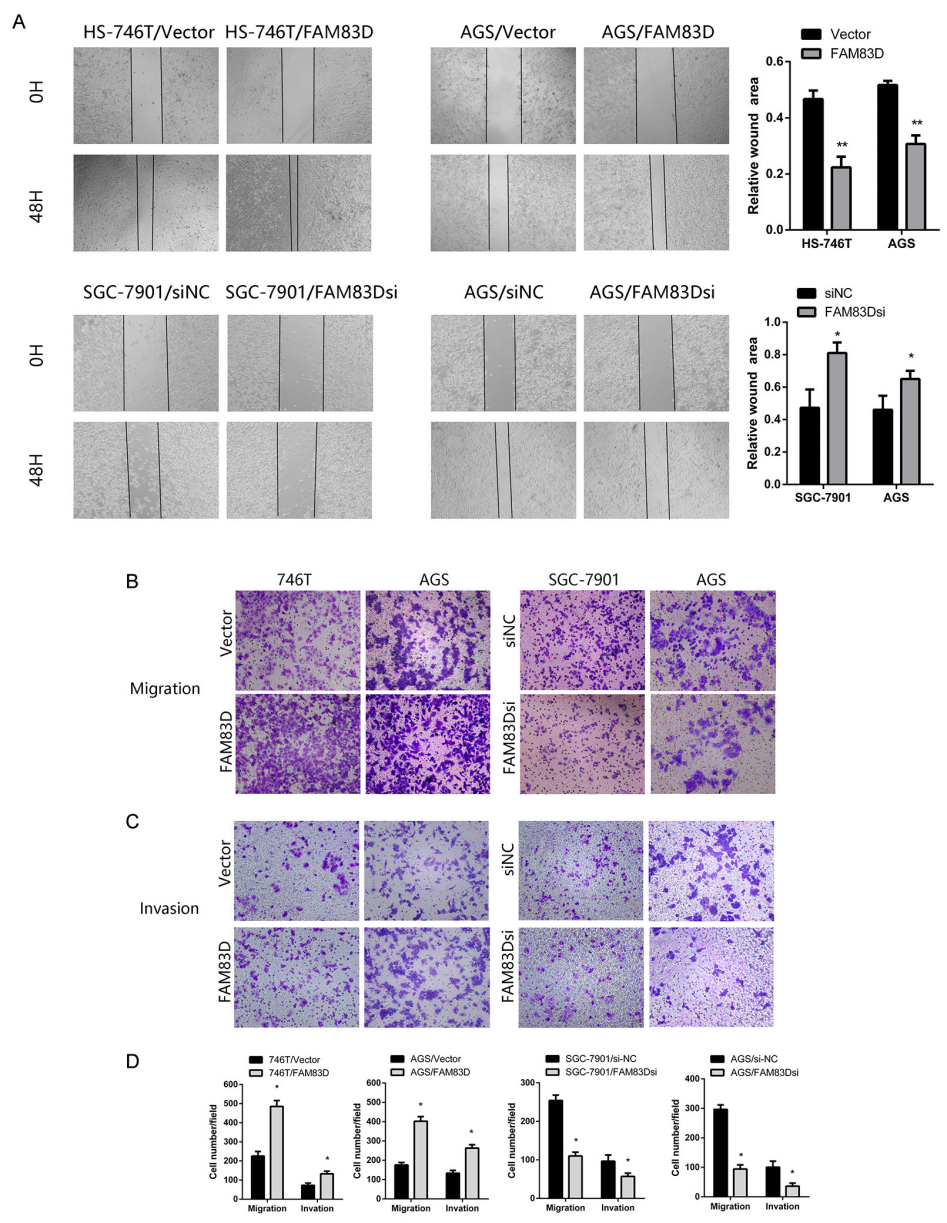
FAM83D enhances migration and invasion of GC cells **(A)** Wound healing assay. Microscopic observations were recorded 0 and 48 hours after scratching the cell surface. **(B and C)** Transwell assay (100x). Representative photographs of migrating or invaded cells on membranes with or without Matrigel are shown. **(D)** Histograms showed the numbers of migration cells and invasion cells. Cells were counted in five randomly selected microscopic fields. Data are means ± SD of three independent experiments, *P < 0.05.

We also investigated if *FAM83D* contributed to the migration and invasion of GC cells. In wound healing assay, we observed that the wound healing area of AGS/*FAM83D* and HS-746T/*FAM83D* cells was markedly smaller than those of AGS/Vector or HS-746T/Vector cells while migration of the AGS and SGC-7901 cells was inhibited by *FAM83D* silencing (Figure 4A, Supplementary Figure 2D). To determine whether ectopic expression of *FAM83D* altered cell migration and invasion, Transwell assays were performed. Knockdown of *FAM83D* attenuated migration (AGS/*FAM83D*si vs. AGS/si-NC: 94.00 ± 14.42 vs. 296.67 ± 15.28 cells per field, P < 0.001; SGC-7901/*FAM83D*si vs. SGC-7901/si-NC: 100.00 ± 10.00 vs. 254.00 ± 14.00 cells per field, P < 0.001) and invasion (AGS/*FAM83D*si vs. AGS/si-NC: 36.33 ± 10.07 vs. 100.33 ± 20.50 cells per field, P < 0.01; SGC-7901/*FAM83D*si vs. SGC-7901/ si-NC: 57.00 ± 8.54 vs. 96.67 ± 16.07 cells per field, P < 0.05). Consistent with these observations, overexpression of *FAM83D* increased cell migration in both AGS and HS-746T lines (AGS/*FAM83D* vs. AGS/Vector: 402.33 ± 24.01 vs. 175.67 ± 12.90 cells per field, P < 0.001; HS-746T/*FAM83D* vs. HS-746T/Vector: 485.00 ± 31.48 vs. 225.67 ± 24.11 cells per field, P < 0.001). Similarly, the numbers of invaded cells were nearly doubled in AGS or HS-746 line, when *FAM83D* was overexpressed (Figure 4B-4D, Supplementary Figure 2E). Next, protein level of *N-cadherin, Vimentin* increased in HS-746T/*FAM83D* cells indicating *FAM83D* overexpression promotes epithelial-mesenchymal transition (EMT) in tumor progression (Supplementary Figure 1D). Together, these results demonstrate that the ectopic expression of *FAM83D* drives the motility and invasion of GC cells.

### Overexpressing *FAM83D* enhances tumor growth and peritoneal dissemination *in vivo*

**Figure 5:**
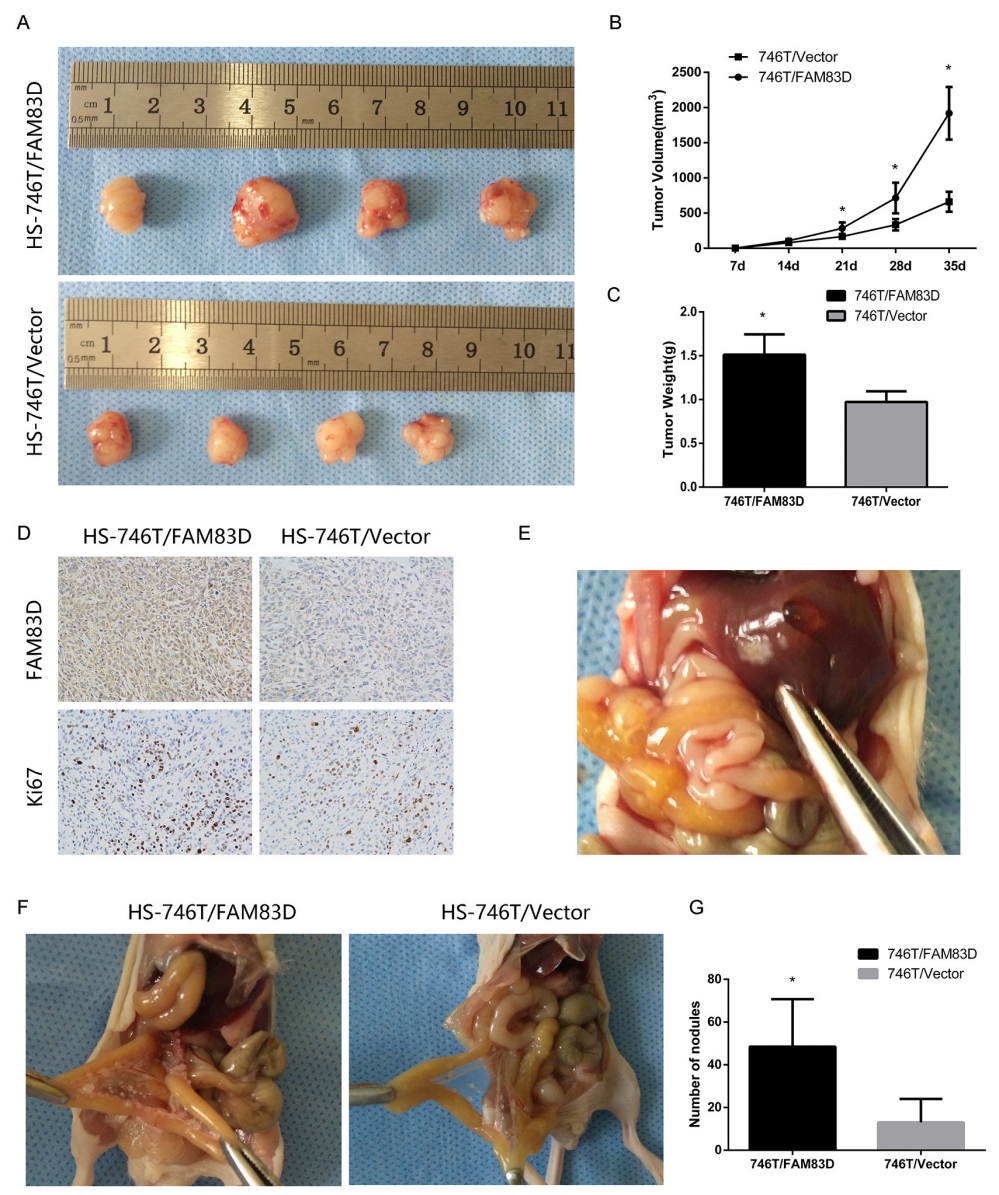
Overexpression of FAM83D promotes subcutaneous tumor growth and peritoneal and other metastases in nude mice **(A)** Photographs of tumors in nude mice derived from HS-746T/FAM83D and HS-746T/Vector cells. **(B)** Growth kinetics of tumors in nude mice. Tumor diameters were measured every 7 days, *P < 0.05. **(C)** The average weight of tumors in nude mice, *P < 0.05. **(D)** Representative photographs of immunohistochemical analysis of FAM83D and Ki-67 antigen in tumors from HS-746T/FAM83D and controls (200x). **(E)** One representative visible nodule on the liver surface. **(F)** Photographs of the peritoneal nodules. **(G)** An average number of peritoneal nodules in each mouse, *P < 0.05.

The xenograft analyses were conducted to evaluate the role of *FAM83D* in the tumorigenicity as well as metastatic progression through peritoneal dissemination in GC. Our data showed that tumor volume or weight in mice injected with the *FAM83D*-overexpressing cells were significantly larger than the control group (P <0.006) (Figure 5A-5C). Moreover, the Ki-67 staining was significantly higher in the HS-746T/*FAM83D* tumors (Figure 5D). Likewise, in peritoneal metastasis mouse models, there were significantly more visible peritoneal nodules in the HS-746T/*FAM83D* group compared with controls (P=0.029) (Figure 5F-5G). In addition, 2 out of 5 mice injected with the HS-746T/*FAM83D* cells developed larger tumors in the liver, but not in the control group (0/5) (Figure 5E). Together, these results illustrate a strong promoting role of *FAM83D* in gastric tumor growth metastasis *in vivo*.

### *FAM83D* knockdown impairs microtubule regrowth

**Figure 6:**
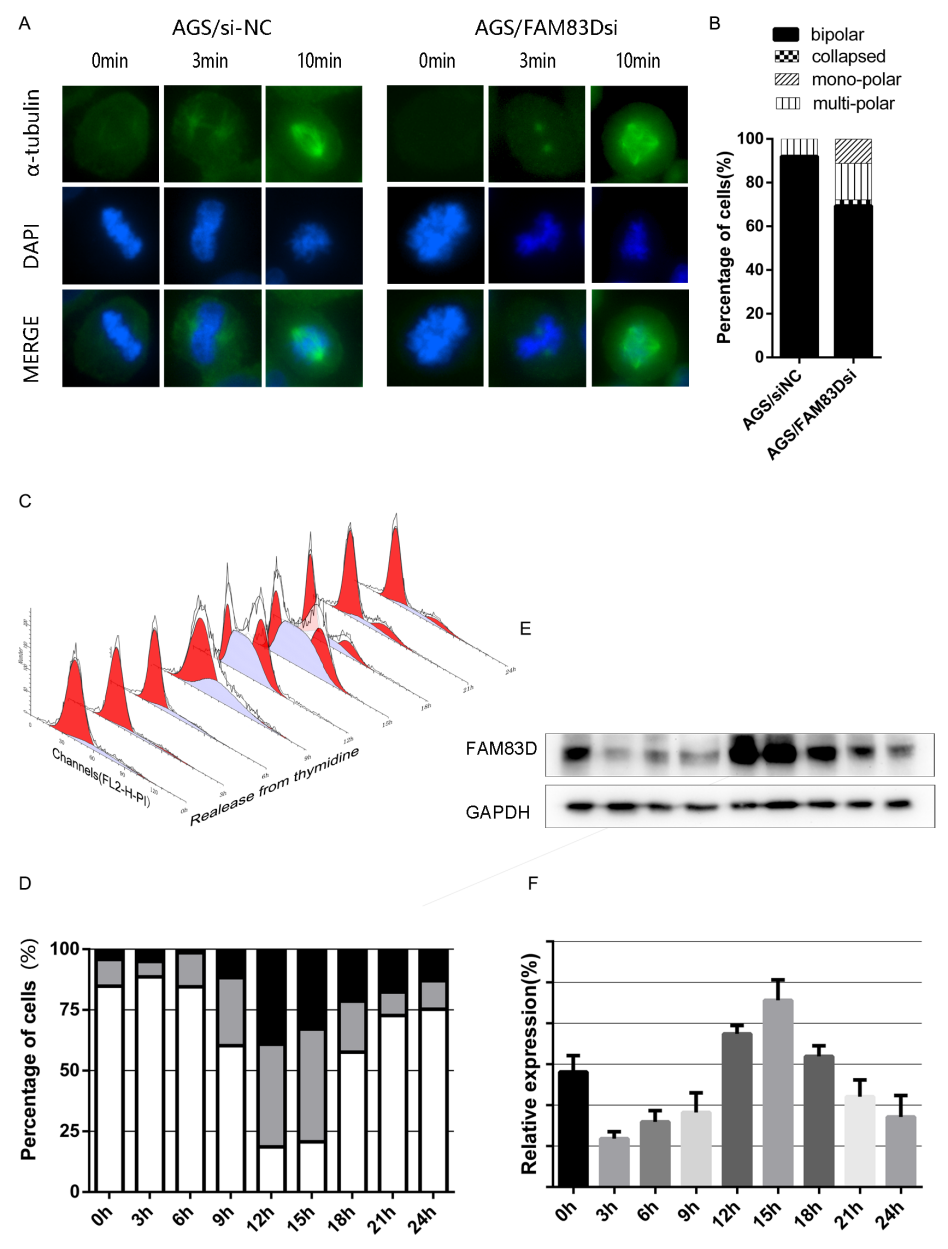
Cold-sensitive microtubules regrowth assay and cell cycle dependent FAM83D expression **(A)** After AGS cells had been transfected with FAM83D siRNA and controls for 36h, cells were incubated at 37°C for 3 or 10 minutes after cold treatment to allow further spindle formation (200x). **(B)** Histograms showed the percentage of spindle abnormalities. **(C and D)** AGS cells were synchronized by double-thymidine block and released. The cell cycle progression was followed by flow-cytometry analysis. **(E and F)** The expression pattern of FAM83D in synchronized AGS cells was analyzed by Western blot.

Cell division requires maintenance of a highly ordered bipolar structure. Thus, a subtle change in non-motor proteins may lead to genetic alteration, which in turns causes cancer or other human diseases. Despite being localized mainly to non-kinetochore microtubules, the *FAM83D* depletion appears to have no effect on K fiber stability [13, 15]. In line with this conclusion, we explored whether cold-sensitive microtubules regrowth was impaired in *FAM83D*-depleted cells. After transfected with siRNA for 36h, cells were treated on ice (4 °C) for 40 minutes to fully depolymerize non-kinetochore microtubules, and then transferred to 37°C for 3 or 10 minutes to allow microtubule regrowth. The intriguing finding demonstrated that microtubule nucleation from centrosomes was not affected as astral microtubules were normally formed from centrosomes after 3 min at 37 °C. However, spindles in AGS/*FAM83D*si cells were frequently abnormal and disorganized after 10 minutes at 37 °C, being mono-polar, multi-polar or collapsed in 11.1%, 16.7% and 2.7% of cases, respectively (Figure 6A-6B). All these data pointed out that lack of *FAM83D* resulted in aberrant spindle formation and maintenance during mitosis. However, referring to cancer issues, ectopic expression of *FAM83D* contributes to tumor progression.

### *FAM83D* expression is cell cycle dependent

The level of *FAM83D* amplification during mitosis has been reported in Hela S3 cells, suggesting a role in mitosis [13]. In order to confirm and further investigate the protein expression pattern of cell cycle progression, we examined cell cycle phase distribution of the cells synchronized with double-thymidine block (Figure 6C-6D). As a reference, population doubling time in ATCC (American Type Culture Collection) website is 20hrs. At various time points after release, cells were harvested, and *FAM83D* protein level was analyzed by Western blot. In parallel, cell cycle distribution was analyzed via flow cytometry by monitoring the DNA content. The data showed that the *FAM83D* protein level was cell cycle correlated, which started to rise in the S phase and peaked in the G2/M phase (Figure 6E-6F). Since FAM83D is a microtubule-associated protein, we have confirmed its cellular localization at different cell cycle stages through fluorescence microscope (Supplementary Figure 1A), which was in corresponding with previous researches [13, 15].

### FAM83D physically binds to a subset of mitotic spindle proteins

**Figure 7:**
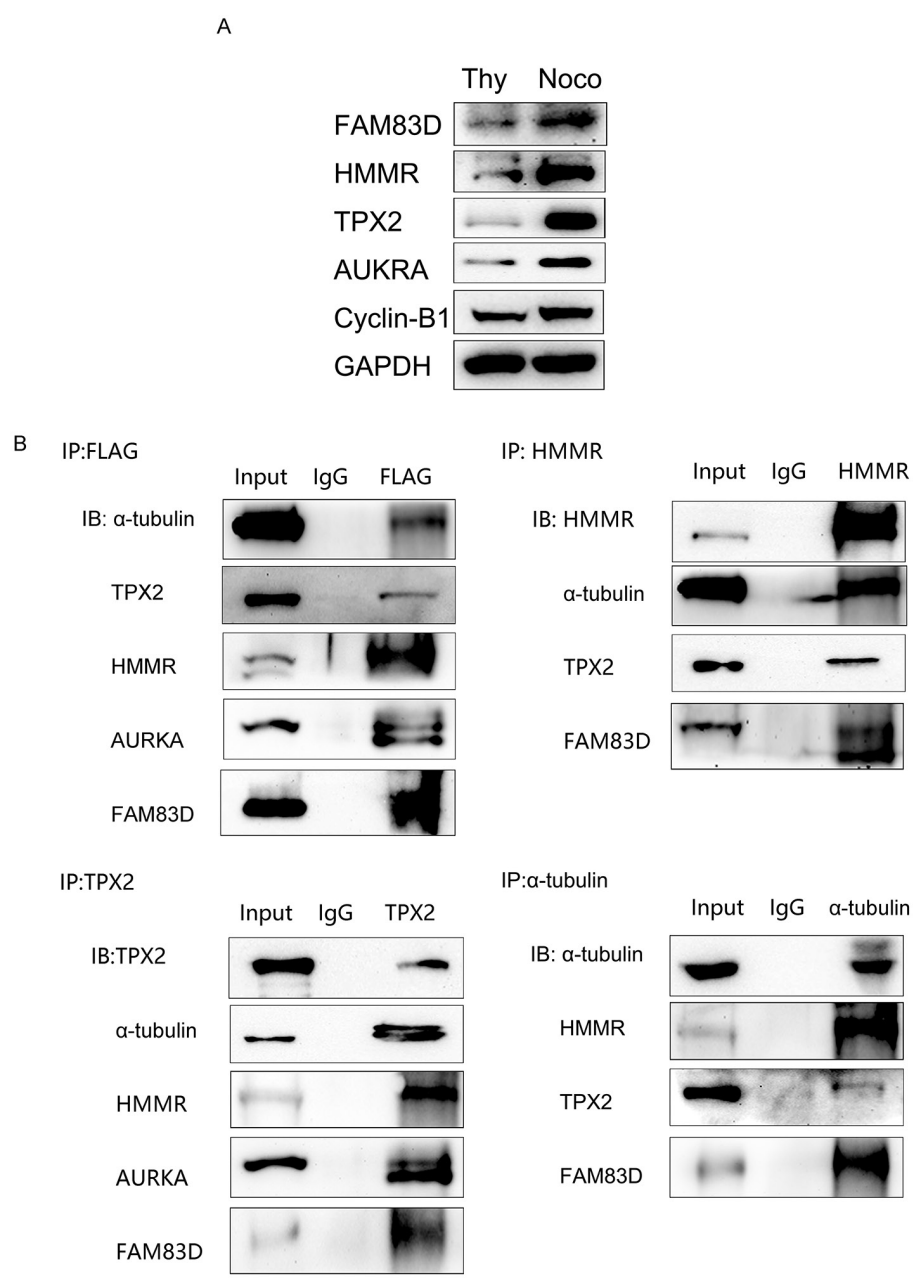
Physical interaction among FAM83D, α-tubulin, HMMR, TPX2 and AURKA **(A)** FAM83D, HMMR, TPX2, and AURKA expression peaked in mitosis compared with G0/G1 phase analyzed by Western blot, with cyclin-B1 as the positive control. **(B)** FAM83D was physically associated with α-tubulin, HMMR, TPX2 and AURKA via co-immunoprecipitation analysis in flag-tagged AGS/FAM83D cells.

The localization of FAM83D at the mitotic spindle raises a possibility that FAM83D may stimulate spindle formation and mitotic progression through protein interaction on the spindle. A recent research analyzing *FAM83D*-coexpressed genes in various tumor types was performed using the Oncomine cancer microarray database, of which, 150 genes were strongly linked with cell cycle and mitosis based on pathway function enrichment, including *AURKA*, *AURKB*, *CCNA2*, *CCNB1-2*, *CDC20*, *CDC25A-C*, *HMMR*, *PLK1*, *TPX2* [35]. Among which, HMMR was found to correlate FAM83D, TPX2 to the spindle, while TPX2 was required for targeting and activating AURKA [15, 19–21]. Based on the previous researches, we hypothesize that FAM83D could bind with HMMR, TPX2, AUKRA on spindle during mitosis and functioned as a complex. *HMMR*, *TPX2* and *AURKA* protein levels peaked in mitosis compared with the G0/G1 phase in AGS cells using nocodazole and thymidine block separately investigated by Western blot (Figure 7A). Our immunoprecipitation analyses showed that the Flag-tagged FAM83D was co-immunoprecipitated with endogenous FAM83D, α-tubulin, HMMR, TPX2 and AURKA in AGS cells, and vice versa. (Figure 7B). These data demonstrate strong physical interactions between FAM83D and a set of spindle-linked proteins, including HMMR, TPX2, and AURKA, in human gastric tumor cells.

## DISCUSSION

Prior to the current study, there was little known about the function of *FAM83D* in GC. Here, we show that *FAM83D* is overexpressed in human gastric tumors, and its overexpression strongly correlates with lymph node metastasis and TNM stage. Our functional analyses also reveal that *FAM83D* overexpression promotes tumor cell proliferation, colony formation, migration, and invasion, as well as tumor growth and metastatic dissemination. Furthermore, we demonstrate that *FAM83D* drives cell cycle progression through the G2/M phase. This function may largely associate with the physical binding between FAM83D and a set of spindle–linked regulators, including HMMR, TPX2, and AURKA. Nevertheless, our study provides evidence for the first time that lack of *FAM83D* leads to aberrant spindle formation. Importantly, our data show that *FAM83D* is essential for maintaining normal MT related event, and confers strong oncogenic activity upon overexpression. Together, our study suggests that *FAM83D* is a crucial driver of gastric tumor growth and metastasis.

Importantly, some previous researches have emphasized an important role of FAM83D in mitosis progression through correlating with other MAPs [13, 15]. Many genes like HMMR, TPX2, and AURKA are microtubule-associated proteins participating in cell division event and tumorigenesis [20–22, 24–27, 29, 36–39]. CO-IP revealed the interaction among FAM83D and three other proteins. Therefore we propose that the association of which gives rise to a novel functional unit with oncogenic properties. Most of the interacting genes, including *FAM83D*, *TPX2*, and *AURKA*, are all located on 20q, which is frequently amplified across many cancer types [7–9]. Mitotic network components such as AURKA/B signaling, FOXM1/PLK1 signaling and E2F/targets of C-MYC are evaluated in most GC subtypes including EBV, MSI, and CIN, indicating the potential role of mitotic genes in GC [40]. This may in part explain the mutual promotion between genomic instability and amplifications of mitotic genes and cell cycle mediators.

Although the detailed mechanism underlying the co-operative function among FAM83D and HMMR/TPX2/AURKA was not clear at this time, it suggests a novel potential candidate therapeutic target complex. Further investigation will be conducted to explore whether the TPX2/AURKA location is FAM83D dependent, whether the other three proteins could affect the FAM83D location, expression, phosphorylation, and function. How FAM83D functions to activate this unit or serves as a scaffold remains to be studied. Furthermore, our study suggests that FAM83D could accelerates the G1/S transition which is also consistent with a recent study on this molecule [6]. However, our observation that *FAM83D* ablation has a minimal suppression on cell apoptosis is deviated from a prior study on breast cancer [10]. These discrepancies are likely attributed to the variation in cancer type or cell line. Therefore, it cannot be ruled out that various pathways and mechanisms *FAM83D* involved in proliferation.

Recently, neoadjuvant intraperitoneal and systemic chemotherapy (NIPS) trials using intraperitoneal paclitaxel are conducted in Asia [41–44]. Paclitaxel induces dysfunctional microtubules in mitosis, which leads to cell death [45]. To explore MAPs such as FAM83D may help to improve treatment outcome. Our further investigation will focus on the effects of FAM83D knockdown on the cytotoxicity of paclitaxel and whether that FAM83D knockdown sensitizes cells to paclitaxel treatment.

Overall, our study has demonstrated that *FAM83D* overexpression drives gastric tumor cell proliferation, migration, invasion as well as tumor growth and metastatic dissemination to peritoneal cavities *in vivo*. Also, *FAM83D* is essential for cell cycle progression. Given the notion that a subtle change in mitotic regulators at protein level could give rise to a great risk of cancer, our study underscores the potential of *FAM83D* as a valuable target for gastric cancer therapy.

## MATERIALS AND METHODS

### Cell lines and cell culture

Human gastric cancer cell lines, including AGS, SGC-7901, NCI-87, MKN-45, HS-746T, and GES-1, were purchased from Shanghai Institutes for Biological Sciences, Chinese Academy of Sciences. Cells were cultured in RPMI-1640 medium containing 10% fetal bovine serum (FBS) at 37 °C in a humidified atmosphere with 5% CO_2_.

### Transfection and analyses of mRNA expression

HS-746T, AGS cells were infected with the Lenti-FLAG-*FAM83D* and control Lenti-Vector purchased from Asia-Vector Biotechnology (Shanghai) Co. LTD and selected with 5 μg/ml blasticidin for 7 days. The siRNAs of *FAM83D* were purchased from Shanghai GenePharma Co. LTD. The SGC-7901 and AGS cells were transiently transfected with siRNAs using Lipofectamine 2000 transfection reagent (Invitrogen), according to the manufacturer’s recommendations (Supplementary Figure 1B-1C). The sequences of siRNAs were listed in Supplementary Table 1. Unless specified, *FAM83D* siRNA duplex number 1 was used for the following transfection.

For qRT-PCR analysis, total RNA isolation were described previously [46]. The primers were listed in Supplementary Table 2.

### Analyses of cell proliferation, colony formation, cell cycle and immunofluorescence

Cell Counting Kit-8 assay was performed as described [46]. For colony formation assay, cells were seeded in 6-well plates at a density of 1000 cells per well. After 7 days, colonies were visualized by crystal violet staining.

Serum starvation was used to induce cell cycle synchronization roughly before cell cycle distribution was analyzed by flow cytometry as described [46]. For analyzing *FAM83D* expression in the cell cycle, AGS cells were synchronized at G0/G1 by double-thymidine block. Briefly, cells were treated with 2mM thymidine (Sigma, USA) for 20 h, followed by culturing in fresh media for 9 h. After that, cells were treated again with thymidine for 16 h and were released from the block by washing in PBS (phosphate-buffered saline) and culturing in fresh media. Samples were taken after block at 0, 3, 6, 9, 12, 15, 18, 21, and 24 hours for flow cytometry analysis and Western blot analysis.

For the immunofluorescence analyses, tumor cells were incubated in 8-well glass slides (Millipore, USA) with primary antibodies against FAM83D (Bioss, CHINA), alpha-Tubulin (Proteintech, USA), followed by incubation with Goat anti-mouse IgG (H+L), FITC conjugate or Goat anti-rabbit IgG (H+L), TRITC conjugate (Proteintech, USA). DNA was stained with 406-diamidino-2-phenylindole.

### Wound healing, transwell migration and invasion assays

Monolayers of cells were plated in 6-well plates and grown until confluent. Scratch wounds were made using sterile 200 μl pipette tips with non-adherent cells and debris removed by PBS washing. The cell motility images were photographed at 0, 48 hours. Cell migration and invasion assays were assessed using transwell chambers (Corning Costar, USA). Especially for the invasion assay, the upper chamber was pre-coated with Matrigel (BD Bioscience, USA) according to the manufacturer’s protocols. 1x10^5^ cells in serum-free medium were added into the upper chamber, and medium with 10% FBS was added to the lower chamber. After cultured for 24h, cells that migrated through the membrane pores to the lower surface of the membrane were fixed with methanol and stained with 0.1% crystal violet for 30 min.

### Immunoprecipitation (IP) and Western blot

Preparation of cell lysates and immunoblotting were conducted as described in a prior study [46]. The primary antibodies used in the current study included those being anti-GAPDH (Proteintech, USA;1:10000), anti-Cyclin-B1 (Cell Signaling Technology (CST), USA;1:1000), anti-FAM83D (Santa Cruz Biotechnology, USA;1:500), anti-TPX2 (Abcam, USA;1:1000), anti-HMMR (Genetex, USA;1:1000), anti-AURKA (Abcam, USA;1:1000) and anti-FLAG (Proteintech, USA;1:5000).

For immunoprecipitation experiments, mitotic cells were incubated with 300nM nocodazole (Sigma, USA) for 24h and collected by shake-off. The cells were then lysed with NP-40 lysis buffer (Beyotime, CHINA)NP-40 Lysis BufferNP-40 Lysis BufferNP-40 Lysis Buffer and pre-cleared for 30 min with protein A/G plus agarose (Santa Cruz Biotechnology, USA). Pre-clear lysate, according to the manufacturer’s instructions, were incubated with primary antibodies overnight at 4 °C on a rocker platform, followed by incubation with 20 μl of re-suspended volume of protein A/G plus agarose for 4h at 4 °C with rotation. Washing was performed five times in 1.0ml lysis buffer, 5min each on a rotator. Bound proteins were released by boiling in 20 μl of Laemmli buffer.

### Immunohistochemistry (IHC) analyses of human tumor tissue microarrays

Human gastric tumor and adjacent noncancerous tissues were obtained from patients undergoing radical gastrectomy at Ruijin Hospital, Shanghai Jiao Tong University School of Medicine (Details in Table 1 ). None of these patients received radiotherapy or chemotherapy prior to the surgery. All tumor tissues were fixed and assembled into paraffin-embedded tissue microarrays. The TNM-stages of patients were determined by the UICC TNM classification. The protocol for the human tissues-related analyses was approved by the Ethics Committee of Shanghai Ruijin Hospital, and all patients were fully informed of the experimental procedures. The IHC staining of human tumor tissues was conducted by use of a rabbit polyclonal antibody against FAM83D (Bioss, CHINA; 1:400), followed staining with secondary antibodies and DAB, according to the protocol given by the manufacture (Dako, USA). The IHC staining was scored as previously described [46].

### Evaluation of tumor growth and metastasis in xenograft models

Four-week-old male BALB/C nude mice were purchased from the Institute of Zoology, Chinese Academy of Sciences of Shanghai and housed in a specific pathogen-free environment. All experiments were performed in accordance with the official recommendations of the Chinese animal community. Briefly, 7x 10^5^ cells in 100μl PBS were injected subcutaneously into the dorsal flank of 8 nude mice or 2x10^5^ cells in 200μl PBS injected into abdominal cavity of 10 mice. All mice were sacrificed after 40 days. Subcutaneous tumor grafts were weighted, and the quantity of nodules present in the abdominal cavity was measured.

### Statistical analyses

All values in the text and figures were presented as mean ± standard deviation (SD). Statistical analyses were performed using Student’s t-test, χ^2^ test (GraphPad Prism 6). A two-tailed t-test at p-value < 0.05 was considered statistically significant.

## SUPPLEMENTARY MATERIALS FIGURES AND TABLES



## Abbreviations

*FAM83D*: family with sequence similarity 83, member D
MAP: microtubule-associated protein
GC: gastric cancer
MT: microtubule
CO-IP: co-immunoprecipitation
HMMR: hyaluronan-mediated motility receptor
AURKA: aurora kinase A
