# Supplementary material for: FAM83D, a microtubule-associated protein, promotes tumor growth and progression of human gastric cancer
Source: Oncotarget. 2017 Aug 10;8(43):74479–93. doi: 10.18632/oncotarget.20157 (PMC5650356; doi:10.18632/oncotarget.20157)
Supplement: Supplementary file 1 [file oncotarget-08-74479-s001.pdf]

FAM83D, A microtubule-associated protein, promotes tumor growth and progression of human gastric cancer

SUPPLEMENTARY MATERIALS

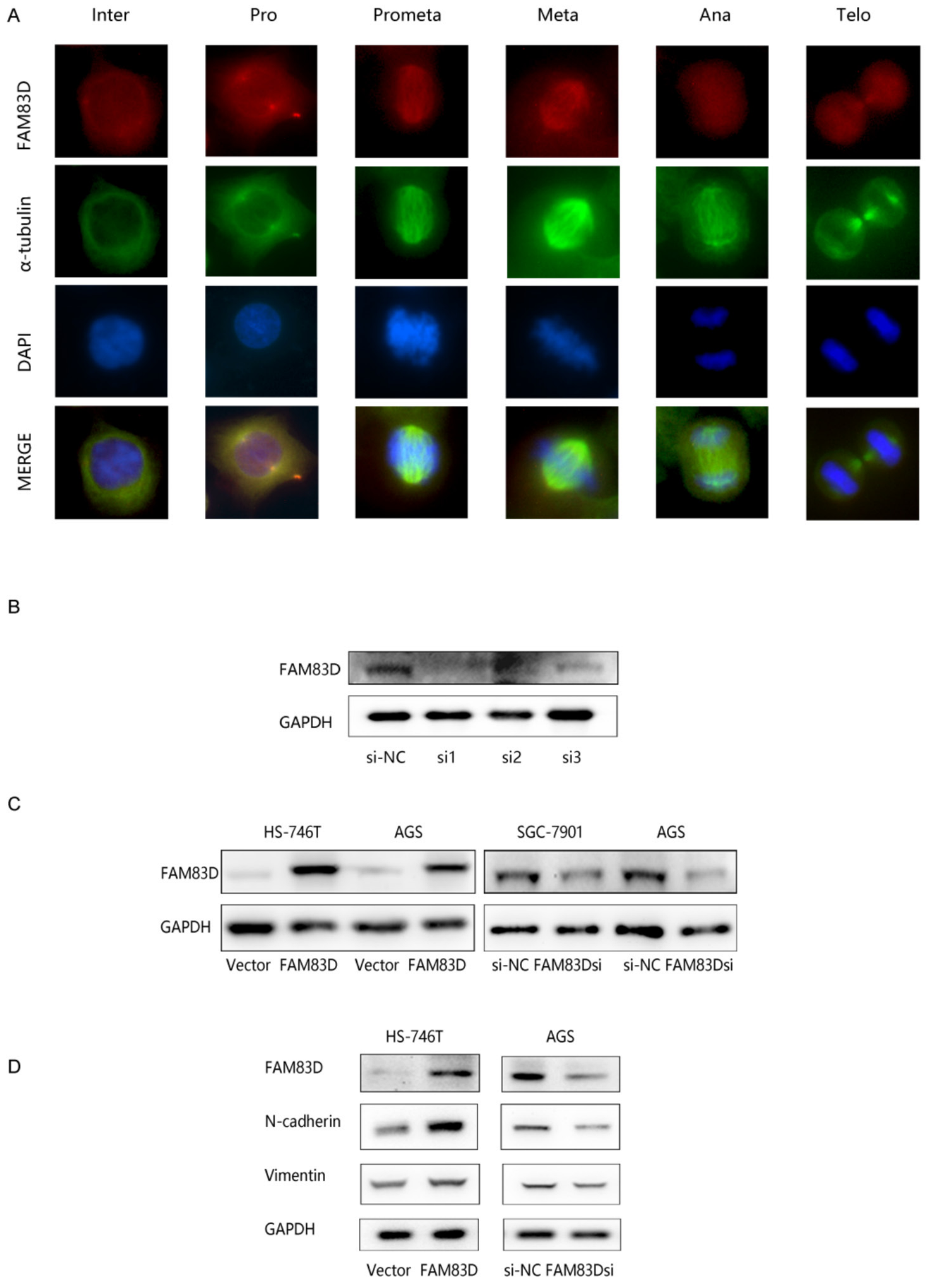

Supplementary Figure 1: (A) Representative photographs of FAM83D location in different stages of mitosis are shown. **(B)** AGS cells transfected with siRNA-1,-2,-3 was analyzed by Western blot. SiRNA-1 was chosen for the following experiments. **(C)** FAM83D protein expression in HS-746T, AGS cells transfected with FAM83D lenti-virus and SGC-7901, AGS cells transfected with siRNA was quantified with Western blot. **(D)** Western blot analysis of N-cadherin and vimentin indicates epithelial-mesenchymal transition (EMT) in FAM83D upregulated cells compared to control cells and knockdown groups.

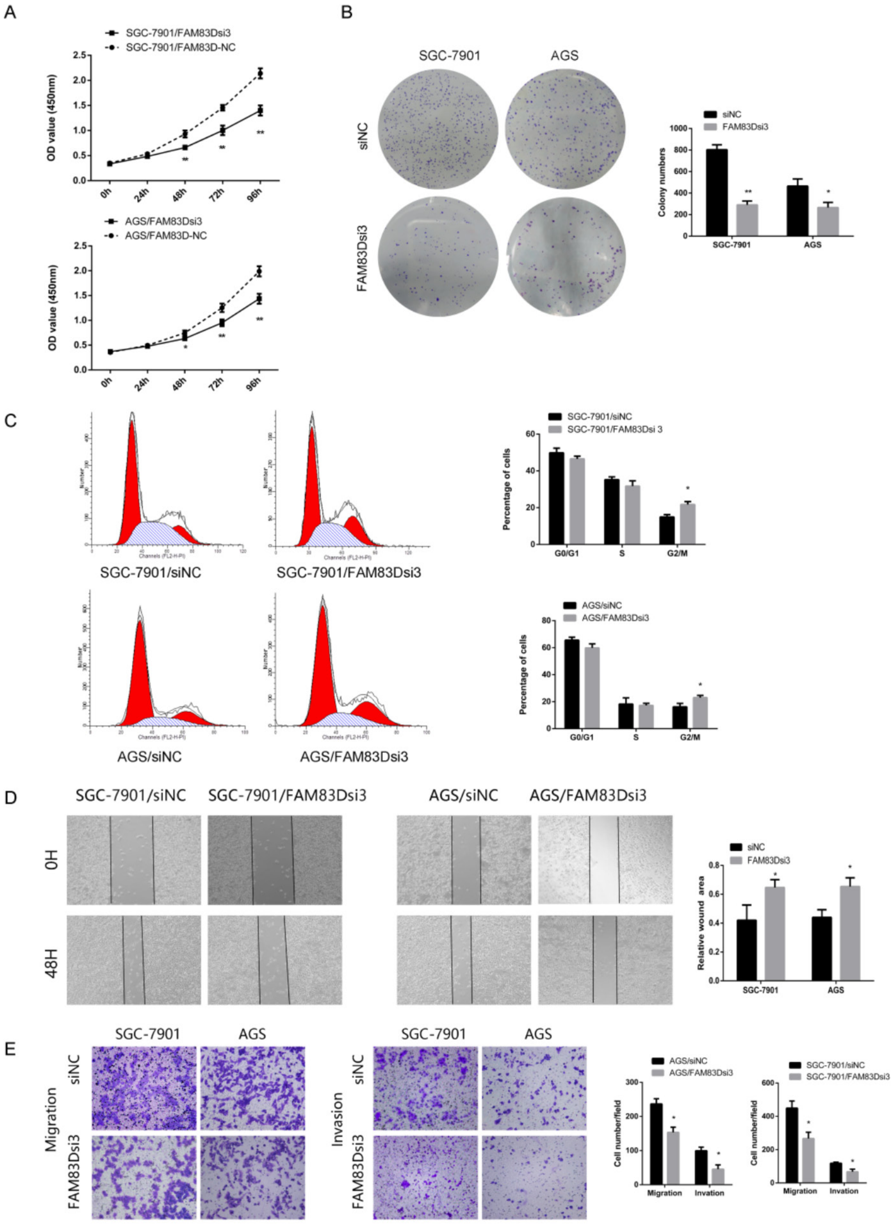

Supplementary Figure 2: Knock down FAM83D by siRNA-3 suppressed GC cell proliferation and metastasis. (A and B) SGC-7901 and AGS cells transfected with FAM83D siRNA-3 and controls were subjected to CCK8 assay. Photographs and histograms of colony formation assay demonstrated the number of colonies, *P < 0.05, **P < 0.01. **(C)** Flow cytometry analysis was performed on SGC-7901 and AGS cells that were transfected with FAM83D siRNA-3 and control siRNA. **(D and E)** Wound healing assay. Microscopic observations were recorded 0 and 48 hours after scratching the cell surface. Transwell assay (100x). Representative photographs of migrating or invaded cells on membranes with or without Matrigel are shown. Histograms showed the numbers of migration cells and invasion cells.

Supplementary Table 1: The sequence of siRNA. 3 siRNA sequences was chosen from published articles

	Sense (5'-3')
FAM83D-si1 [1]	GCAGUAACUUGGUAAUUCUTT
FAM83D-si2 [2]	CCUCUACUGUUAGUGAGGAdTdT
FAM83D-si3 [3, 4]	CCAGGATAGCAAGCTCTCAAA

1. Liao W, Liu W, Liu X, Yuan Q, Ou Y, Qi Y. Upregulation of FAM83D affects the proliferation and invasion of hepatocellular carcinoma. Oncotarget. 2015; 6: 24132–47. https://doi.org/10.18632/oncotarget.4432.
2. Wang D, Han S, Peng R, Wang X, Yang X, Yang R, Jiao C, Ding D, Ji G, Li X. FAM83D activates the MEK / ERK signaling pathway and promotes cell proliferation in hepatocellular carcinoma. Biochem Biophys Res Commun. 2015; 458: 313–20. https://doi.org/10.1016/j.bbrc.2015.01.108.
3. Santamaria A, Nagel S, Sillje HW, Nigg EA. The spindle protein CHICA mediates localization of the chromokinesin kid to the mitotic spindle. 2008; 18: 723–9. https://doi.org/10.1016/j.cub.2008.04.041.
4. Dunsch AK, Hammond D, Lloyd J, Schermelleh L, Gruneberg U, Barr FA. Dynein light chain 1 and a spindle-associated adaptor promote dynein asymmetry and spindle orientation. J Cell Biol. 2012; 198: 1039–54. https://doi.org/10.1083/jcb.201202112.

Supplementary Table 2: Primers

		Sense(5'-3')
FAM83D	Forward	CTCTTCGGGCACCTACTTCC
	Reverse	ACCACTGCAATCACCTCTCG
GAPDH	Forward	CCCATCACCATCTTCCAGGAG
	Reverse	CTTCTCCATGGTGGTGAAGACG
